# An Entropy-Based Machine Learning Algorithm for Combining Macroeconomic Forecasts

**DOI:** 10.3390/e21101015

**Published:** 2019-10-19

**Authors:** Carles Bretó, Priscila Espinosa, Penélope Hernández, Jose M. Pavía

**Affiliations:** 1 Department of Economic Analysis, Universitat de València, Avda. Tarongers s/n, 46022 Valencia, Spain; carles.breto@uv.es; 2 Department of Applied Economics, University of Valencia, Avda. Tarongers s/n, 46022 Valencia, Spain; priscila.espinosa@uv.es; 3 ERI-CES, UMICCS, Department of Economic Analysis, University of Valencia, Calle Serpis 29, 46022 Valencia, Spain; penelope.hernandez@uv.es; 4 UMICCS, Department of Applied Economics, Universitat de València, Avda. Tarongers s/n, 46022 Valencia, Spain

**Keywords:** maximum-entropy inference, Kullback–Leibler, combining predictions, GDP, averaging

## Abstract

This paper applies a Machine Learning approach with the aim of providing a single aggregated prediction from a set of individual predictions. Departing from the well-known maximum-entropy inference methodology, a new factor capturing the distance between the true and the estimated aggregated predictions presents a new problem. Algorithms such as ridge, lasso or elastic net help in finding a new methodology to tackle this issue. We carry out a simulation study to evaluate the performance of such a procedure and apply it in order to forecast and measure predictive ability using a dataset of predictions on Spanish gross domestic product.

## 1. Introduction

This paper applies a Machine Learning approach with the aim of providing a single aggregated prediction from a set of individual predictions. Departing from the well-known maximum-entropy inference methodology, a new factor capturing the distance between the true and the estimated aggregated predictions presents a new problem. To tackle the issues posed by this additional factor, one can look at machine learning (ML) algorithms like ridge regression, lasso or elastic nets. By doing so, the main contribution of this paper is a novel algorithm that combines classic maximum-entropy inference with machine learning and regularization principles by applying a penalty when the aggregated forecast fails to match the forecast target. Via a simulation exercise, we assess the performance of the algorithm and compare it against the naive approach in which aggregated predictions are built as averages of individuals predictions. We also apply this algorithm to a dataset of predictions on Spanish gross domestic product to produce optimal weights that are then used to produce predictions, the predictive ability of which is also evaluated.

Nowadays, there is an increasing number of prospective sources and methods stating a wide variety of forecasts for a given economic variable. The traditional methods for combining forecasts are based on the relative past performance of the forecasters to be combined. However, the number of forecasters has increased considerably over recent years, with the new ones not having had enough time to sufficiently demonstrate their predictive ability, an issue relevant in Economics.

The convenience of combining individual results to obtain a single aggregated prediction is not only problematic in Economics. In Physical Theory, understood as Statistical Mechanics, the seminal works of Jaynes ([1,2]) provide the connection with Information Theory that suggests a constructive method for setting up probability distribution with partial knowledge. Another reason why an Information Theory approach could be a more appropriate way of tackling the problem of the prediction aggregation is an informational matter. Rational expectation says that experts should converge eventually to the true prediction. After a long but successful learning process, experts should make similar predictions. Therefore, a uniform distribution over the set of predictions should be the ultimate combination of predictions. Such a distribution maximizes its entropy.

The machine learning literature on combining forecasts is vast and includes among others the approaches of bagging [3], boosting [4,5] or neural network blending [6]. In the field of economics, combining forecasts has a long tradition and is still an active area (see, e.g., Refs. [7,8,9,10]). Prediction combination in order to forecast gross production represents also an active subfield of research (e.g., Refs. [11,12,13,14,15,16]). The ASA/NBER business outlook surveys started producing composite economic forecasts on 1968 shortly after Ref. [17] commented on the advantages of averaging several forecasts of gross production (as pointed out in Ref. [18]).

From the classic theory, the combination of individual results to obtain a single aggregated prediction consists on a vector of weights that calibrates different degrees of expert ability. Several alternatives can be considered for the combination of forecasts involving different degrees of sophistication. For instance, Ref. [19] considers a minimization of variance-covariance; Ref. [20] offer a method to compute the weights in order to minimize the error variance of the combination. Another method called the regression methods by Ref. [21] interprets the coefficient vector of a linear projection as the corresponding weights. This line of research takes into account the same optimization problem by changing the restriction conditions. We present the benchmark model for the optimization problem of the aggregation of prediction under the perspective of Information Theory. This model activates the criterium of Kullback–Leibler distance to determine the weights of the aggregation of prediction. The nature and objectives of the above problem consists of combining the predictions trying to keep constant (uniform) the knowledge provided by each of them and verifying the true prediction.

Under this perspective, a second approach, the Machine Learning technique, presents a second optimization problem. We draw inspiration from some machine learning algorithms to suggest a specification that combines both objectives: the relative distance expression and the constraints part related to the true prediction. We propose a new specification that also introduces temporal parameters related to an arbitrary temporal structure. Parameters that weight each of the divergences between the aggregation of the predictions and the true predictions. The resulting optimization problem resembles that of regression with regularization [22] and we propose solving it using nested cross-validation [23].

Empirical features of the proposed algorithm are illustrated using a dataset of predictions on Spanish gross domestic product (GDP). The dataset used in this application comes from Fundación de las Cajas de Ahorro, FUNCAS. This is a rich dataset with a sufficient number of institutions making predictions to allow the use of the proposed algorithm. Using this dataset, the proposed algorithm produces optimal weights which are then used to produce both predictions and the predictive ability. Although the dataset does not allow us to disentangle clear differences between the proposed algorithm and a naive forecast, the algorithm is robust in the sense that selecting predictions made in either July or December leads to similar results and interpretations.

The differences between the proposed algorithm and the naive forecast are further explored in a simulation study. Such a study reveals that the proposed algorithm becomes more suitable than the simpler, naive overall average as the length of the target time series increases, as the number of forecasting institutions decreases and as the institutions with predictions sharper than the rest become fewer in number and depart more from the rest.

The paper is organized as follows. In Section 2 we present the model. In Section 3 we introduce the Machine Learning algorithm applied to the maximum-entropy inference problem. In Section 4 the above algorithm is applied to a dataset of predictions on Spanish gross domestic product and in Section 5 assessed via a simulation exercise. Section 6 presents the concluding remarks.

## 2. Model

This section presents first the benchmark model for the optimization problem of the aggregation of predictions under the perspective of Information Theory. This model activates the criterium of Kullback–Leibler distance to determine the optimal weights of the aggregation of predictions. A second approach, the machine learning technique, provides the second model. Finally, the relationship between both approaches is described.

### 2.1. Benchmark Maximum-Entropy-Inference (MEI)

Given a set of agents *I*, let ${\left\{y_{i,t}\right\}}_{i\in I,t\geq 0}$ be forecasts for an economic variable at time *t* made at a prior time. We consider the combination of the individual results or weighted by a vector of parameters for each possible forecast denoted by $\omega_{i}$. The weights $\omega_{i}$ are interpreted as the degrees of expertise for every agent i∈I. By assuming a non-degenerate distribution of weights, the true prediction at time *t* is denoted $a_{t}$, which verifies $\sum_{i\in I}\omega_{i}y_{i,t}=a_{t}$. The first problem we tackle is to find out the weights $\omega_{i}$ such that the true prediction fits the aggregation of predictions.

A parallel problem that we consider is the entropy maximization of the distribution of ${\left\{\omega_{i}\right\}}_{i\in I}$ subject to the true value coinciding with the aggregation of predictions for all possible temporal horizon *t*. This optimization problem is expressed as follows:

$$\begin{array}{ccc} & \max_{\omega_{i}}\sum_{i\in I}\omega_{i}\log\omega_{i}^{-1} & \\ \mathrm{subject}\mathrm{to} & \sum_{i\in I}\omega_{i}=1 & \omega_{i}\geq 0,\\ & \sum_{i\in I}\omega_{i}y_{i,t}=a_{t} & \mathrm{for}t\geq 0 \end{array}$$

This methodology known as maximum-entropy inference is equivalent to the problem of finding out a non-negative distribution of weights ${\left\{\omega_{i}\right\}}_{i\in I}$ that minimizes the Kullback–Leibler-distance between such a distribution and the uniform distribution over the set of agents, that is, $\frac{1}{\left|I\right|}$. The Kullback–Leibler distance between two distributions *p* and *q* is defined as $K\left(p,q\right)=\sum_{x}p\left(x\right)\log\frac{p(x)}{q(x)}$. Notice that the Kullback–Leibler distance is always non-negative but it is not a proper distance since it neither verifies the symmetric nor the triangular properties. This approach based on the Kullback–Leibler-distance assures a non biased outcome over the set of agents.

Formally,

(1) $$\min_{\omega_{i}}\sum_{i\in I}\frac{1}{\left|I\right|}\log(\omega_{i}{\left|I|\right)}^{-1}$$

$$\begin{array}{ccc} \mathrm{subject}\mathrm{to} & \sum_{i\in I}\omega_{i}=1 & \omega_{i}\geq 0,\\ & \sum_{i\in I}\omega_{i}y_{i,t}=a_{t} & \mathrm{for}t\geq 0 \end{array}$$

To solve this, we start with the Lagrangian of the above problem. It should be noted that the cardinality of variables compared with the set of restrictions may be not enough to guarantee a unique solution, even the existence of a solution. Despite being able to characterize a set of possible weights that minimize the relative distance, it may not fit the true prediction condition.

This issue is well recognized in the literature, since the complexity of finding a proper solution increases with the cardinality of the parameters and conditions; hence, it is necessary to use numerical algorithms to find out (if it exists) the set of candidates of solution.

In order to reduce the complexity of the problem, we can consider a new parametrization of ${\left\{\omega_{i}\right\}}_{i\in I}$. In particular, one can reparameterize $\omega_{i}=\frac{e^{x_{i}}}{\sum e^{x_{i}}}$ for $x_{i}\in R$. This guarantees that $\sum_{i}\omega_{i}=1$ with $\omega_{i}>0$ while simplifying the optimization problem by reducing the number of constraints from three to one.

Another way to approach the original problem is to allow a balance between the two restrictions written in the Lagrangian. On the one hand, the distribution of weights should minimize the relative entropy, on the other hand, this system should generate an aggregation of predictions as close as possible to the true prediction.

### 2.2. Machine-Learning-Inference (MLI)

The nature and objectives of problem (1) consists of (i) combining the predictions of a set of institutions, (ii) trying to keep constant (uniform) the knowledge (the information) provided by each of them and (iii) verifying (approaching as much as possible) a set of restrictions. Under this perspective, we draw inspiration from some machine learning algorithms, such as ridge, lasso or elastic net (in ridge, lasso or elastic net the goal is to minimize a distance, keeping under control the number of parameters of the model to avoid overfitting and all this is controlled by a parameter that allow to rescale or determine the relative importance of each source error function) to propose a specification that combines both objectives: the relative distance expression and the constraints part related to the true predictions. We propose a new specification, which also introduces the parameters $\delta_{t}$ related to an arbitrary temporal structure (for example, each of those parameters may depend on the time distance of the restriction to the forecasted period or on the certainty available about the corresponding constraint value), with a parameter λ that weights the restrictions imposed by the distance between the aggregation of the predictions and the true predictions. It is possible to consider a family of norms since the problem is in $R^{n}$ and we are under a normed vector space.

Putting together both expressions and being mindful that the parameters $\left\{\omega_{i}\right\}$ are parametrized, we formalize the minimization problem under a machine learning perspective as:

(2) $$\min_{x_{i}}\sum_{i\in I}\frac{1}{\left|I\right|}\log(\omega_{i}{\left|I|\right)}^{-1}+\lambda \sum_{t}\delta_{t}\left|\right|\sum_{i\in I}\omega_{i}y_{i,t}-a_{t}\left|\right|$$

The connection of the proposed specification to the machine learning literature stems from the form of the objective function (Equation (2)) and its two summands. The first one refers to the divergence of Kullback–Leibler: $\sum_{i\in I}\frac{1}{\left|I\right|}\log(\omega_{i}{\left|I|\right)}^{-1}$. The second one corresponds (resembles) to a flexible regularization term: $\lambda \sum_{t}\delta_{t}\left|\right|\sum_{i\in I}\omega_{i}y_{i,t}-a_{t}\left|\right|$.

Lambda (λ, hereafter) is a penalty parameter to choose weights that minimize the divergence of Kullback–Leibler to a uniform distribution and penalize the magnitude of the deviation of the weighted prediction from the observed value. On the one hand, when λ is equal to 0 there is no past prediction penalty and the result is equivalent to the classic model without temporal restrictions. On the other hand, when λ grows the breach of the temporal restrictions is gaining weight and dominates Equation (2). In this latter case, the problem may be thought of as a weighted regression problem but with the coefficients restricted to being positive and to adding up to one and without showing the drawbacks of traditional procedures when the number of forecasters is larger than the number of temporal restrictions.

The delta parameters (δ, hereafter) are an improvement measure for the magnitude of the importance that λ gives to the breach of the restrictions. In other words, δ weights the relative importance to the restriction from one year to another.

### 2.3. From Maximum-Entropy Inference to Machine Learning Inference

Problem (1) indeed shares the same essence as the minimization of problem (2). The first problem is a constrained optimization problem and the second one incorporates this restriction to the objective function. The methodology of solving problem (1) is by the method of the Lagrange multipliers. Specifically, the constrained problem is converted into a structural form with both the objective and the constrained conditions together multiplied by parameters depending on the set of restrictions. Solving the first order conditions of the Lagrangian function, the optimum is derived. The Lagrangian for (1) is written as:

$$\begin{array}{cc} L= & \sum_{i\in I}\frac{1}{\left|I\right|}\log(\omega_{i}{\left|I|\right)}^{-1}+\sum_{t}\lambda_{t}\left(\sum_{i\in I}\omega_{i}y_{i,t}-a_{t}\right) \end{array}$$

It should be noted that the solution ${\left\{\left(\omega_{i}^{*},\lambda_{t}^{*}\right)\right\}}_{\left(i\in I,t\right)}$, if it exists, pushes down to 0 the second part of the Lagrangian since the restrictions must hold and moreover minimize the relative distance.

Let us now assume a family of problems denoted by P(λ). Fixing λ we have the following minimization problem:

(3) $$\min_{x_{i}}\sum_{i\in I}\frac{1}{\left|I\right|}\log(\omega_{i}{\left|I|\right)}^{-1}+\lambda \sum_{t}\delta_{t}\left|\right|\sum_{i\in I}\omega_{i}y_{i,t}-a_{t}\left|\right|$$

When the norm is the absolute distance and $\lambda_{t}=\lambda \delta_{t}$, both problems, (3) and (1) coincide. If a solution in the former problem (3) exists, then such a solution is a candidate for the later problem (1) for the specific $\lambda_{t}$. Only the restrictions may not be satisfied in problem (3) if this distortion allows the reduction of (if it is possible) the relative entropy with the uniform distribution. Therefore, under the assumption of existence of solution, both problems will offer the same class of solution.

The consideration in the optimum allows us to consider addressing problem (3) from another perspective when in fact problem (1) has no solution or it is too complex to find. The algorithms and structural forms borrowed by machine learning could be a way to approach the solution from a machine learning framework.

## 3. Algorithm

This section proposes an algorithm to deal with problem (3). The proposed algorithm finds a solution to problem (3) analogously as they do well-understood regularization, machine-learning algorithms. The main steps of the algorithm are splitting the data into training, validation and test sets and choosing the penalty coefficient, λ, via cross-validation on validation sets. The parameters $\delta_{t}$ are exogenous. Following this, the algorithm prediction error can be computed on the test sets and the $x_{i}$ values estimated using the whole set after addressing the λ and δ parameters, bearing in mind that the parameters $\left\{\omega_{i}\right\}$ are parametrized. The estimated values are finally used as weights to combine the individual predictions.

For cross-validation, we follow the time-series machine-learning literature and propose the use of rolling-origin evaluation [24], also known as rolling-origin-recalibration evaluation [25]. These are forms of nested cross-validation, which should give an almost unbiased estimate of error [23]. Once the number of institutions (forecasters) that we could be used to properly define the training, validation and test sets are selected, we can start to solve the optimization problem. As we will have already noticed, the institutions must be the same in the training, testing and validation sets. If this condition is not fulfilled, the problem will not be well defined. To solve this issue, in our application (see Section 4), the dimensionality of the initial data bank was reduced from 21 to around 10 forecasters satisfying the condition of existence of data for the three phases. This gives us three sets of data sampling with around 10 institutions for each phase.

As a possible specification we select one of the possible options, we consider the quadratic norm, a ridge regression, in the objective function and add a parameter λ and the δ’s that characterize the slackness of the process. For simplicity we use $\delta_{t}=1$, where we give equal importance to all restrictions. Different values of λ, from a grid of values, are tested to find the optimum that minimizes the divergence and penalizes the combinatorial prediction with respect to the observed value.

The steps of the proposed algorithm are described in detail in Algorithm 1. The output of the algorithm is a prediction for period T+1 denoted by ${\hat{a}}_{T+1}$. The requirements to apply this algorithm are: (i) the three dataset splits mentioned above (training, validation and test), (ii) a set of discrete values of λ between 0 and infinity, (iii) a set of discount values δ emphasizing the λ parameter, and (iv) a prediction error function. The algorithm solves the optimization problem on the training subset for each of the different values of λ and δ. Once the optimization problem is solved, we get a set of prediction errors on the validation set, as many as values for λ. Subsequently, through cross-validation, we make the selection of the λ that minimizes this prediction error. Thanks to this selection, it is possible to obtain the best penalty in terms of prediction error. Once the best λ is obtained, we apply the algorithm on the test set and evaluate its performance. We get the $\omega_{i}$ that minimize the objective function and a measure of its prediction error.

**Algorithm 1:** Machine learning based entropy
**1** 
**input:**
**2** Forecast data made by institution *i* for year *n*, $\left\{y_{i,n};i\;\mathrm{in}\;1\;:\;I,n\;\mathrm{in}\;1\;:\;N+1\right\}\;\left(N\geq 2\right)$**3** Realized values, $a_{1:N}$**4** Set of penalty coefficients, $\left\{\lambda_{j},j\;\mathrm{in}\;1\;:\;J\right\}$
**5** Set of discount coefficients $\left\{\delta_{t,T},t\;\mathrm{in}\;1\;:\;(N-1),T\;\mathrm{in}\;2\;:\;N\right\}$**6** Forecast error function *f***7** 
**output:**
**8** Prediction ${\hat{a}}_{N+1}$**9** 
**Pseudocode:**
**10** For *n* in 2:N**11**    For *j* in 1:J**12**       Solve for weights using the training subset $y_{1},\ldots ,y_{n-1}$:**13**          Set $\omega_{i,n,j}={\mathrm{argmin}}_{\left\{\omega_{i}\right\}}\sum_{i\in I}\frac{1}{\left|I\right|}\log(\omega_{i}{\left|I|\right)}^{-1}+\lambda_{j}\sum_{t=1}^{n-1}\delta_{t,n}\left|\right|\sum_{i\in I}\omega_{i}y_{i,t}-a_{t}\left|\right|$**14**       Determine the forecast error using the validation set $y_{n}$:**15**          Set $e_{n,j}=f\left(a_{n},\sum_{i\in I}\omega_{i,n,j}y_{i,n}\right)$**16**   End For**17** End For**18**    Set $j^{*}={\mathrm{argmin}}_{j}{\left(N-1\right)}^{-1}\sum_{t=2}^{N}e_{t,j}$**19**    Set $\lambda^{*}=\lambda_{j^{*}}$**20** Solve for weights using $\lambda^{*}$ and the full data set:**21**    Set $\omega_{i}^{*}={\mathrm{argmin}}_{\left\{\omega_{i}\right\}}\sum_{i\in I}\frac{1}{\left|I\right|}\log(\omega_{i}{\left|I|\right)}^{-1}+\lambda^{*}\sum_{t=1}^{N}\delta_{t,N}\left|\right|\sum_{i\in I}\omega_{i}y_{i,t}-a_{t}\left|\right|$**22** Set ${\hat{a}}_{N+1}=\sum_{i\in I}\omega_{i}^{*}y_{i,N+1}$


## 4. Data Analysis

A dataset of predictions on Spanish gross domestic product is used to illustrate empirical features of the proposed algorithm. The proposed algorithm produces optimal weights $\omega_{i}^{*}$ (Table 1) that are used to produce predictions ${\hat{a}}_{T+1}$ (Table 2), the predictive ability of which can be assessed. The predictive ability of the proposed algorithm for this dataset is similar to that of alternative naive forecast algorithms, in agreement with the simulation exercise of Table 3.

The dataset used in this application comes from the Fundación de las Cajas de Ahorro, FUNCAS. The sample covers the economic predictions of different institutions from 2000 to 2018. The selected sample contains a total of 21 institutions: Analistas financieros, Asesor, Bankia, BBVA, Caixabank, Cámara de Comercio de España, CatalunyaCaixa, CEEM-URJC, Cemex, CEOE, CEPREDE-UAM, ESADE, Funcas, ICAE-UCM, IEE, Instituto de Macroeconomía y Finanzas (Universidad CJC), Instituto Flores de Lemus, Intermoney, Repsol, Santander, Solchaga Recio & asociados). Each agency makes two predictions a year, in July and December for both the current and the following year. Therefore, each year is predicted by each agency up to 4 times. FUNCAS prediction panels are very well known with a prominent experience in economic research and for their thorough work in collecting forecasts at the regional and national levels. In addition, FUNCAS provides such information for free (see www.funcas.es).

For this data analysis, a quadratic forecast error function $f\left(x,y\right)={\left(x-y\right)}^{2}$ and the following algorithmic parameter values have been used: $\lambda \in \{1\times 10^{-4},2\times 10^{-4},\ldots ,8\times 10^{15},9\times 10^{15}\}$ and $\delta_{t,T}=1$ for all t,T. The optimization problems have been solved using the free software R version-3.6.1 [26] and the optimization algorithms available in the nloptr library, which serves as an interface for the NLOPT library [27]. NLOPT algorithms can be global or local and based on derivatives or gradient free and include, for example, the augmented Lagrangian algorithm, which uses subsidiary local optimization algorithms. All optimizations have been initialized with a uniform starting point.

To help illustrate the application of the algorithm, Table 1 and Table 2 focus on the subset of the full dataset that only includes forecasts for each given year made in July of that same year. Alternative restrictions of the full dataset are possible, for example, forecasts for each year made in December of that same year or forecasts for each year made in July of the previous year. Such alternative restrictions lead to similar key features regarding predictive ability and optimal weights as are described below.

Key features of the optimal weights $\omega_{i}^{*}$ output by the proposed algorithm included in Table 1 are weight variation across years and across institutions, variations that can be substantial but also reveal some consistencies. The years for which Table 1 reports optimal weights are 2002 through 2018. For the first two prediction years, all weights are negligible except for one, with that single key institution representing about 10% of the number of institutions. For the remaining fifteen years, weights spread out producing 20% to 60% of key institutions. Institutions range from those receiving large optimal weights (e.g., CatalunyaCaixa with 100% on 2002–2003 or IEE and ICO with about 75% on 2004 and 2006 respectively) to those receiving negligible weights. Some institutions are not considered in some years. Of the initial 21 institutions in the full dataset, only 13 produced forecasts from 2000, of which only 9 were still producing forecasts by the end of the sample. Considering years and institutions jointly gives two institution groups: institutions with strikes of substantial weights (e.g., using 25% as threshold: CatalunyaCaixa, IEE and ICO) and the rest of institutions.

Some key factors to assess the predictive ability of predictions ${\hat{a}}_{T+1}$ made using the proposed algorithm included in Table 2 have been varied as parameters in the simulation study. The simulation study considers multiple combinations of different parameters (Table 3). A combination of parameters that resembles the features in the data could be: (i) 40% of key agents, given that about half of the estimated optimal weights in Table 1 are non-negligible , i.e., ≥4% (note however that the fraction of non-negligible weights grows substantially over time in the data while it remains constant in the simulation study); (ii) 10 forecasting agents, given that the number of agents decreases from 13 on 2000 to 9 on 2018; and (iii) a sample size of T=20 years, with the data covering nineteen years (2000–2018). According to the simulation study, such combination of parameters seems to have potential for favoring either the naive or the proposed algorithm depending on the degree of variability between predictions. A variability of SD=0.2 might be reasonable for the data, since predictions for a year are made in July of that same year. This amount of variability produced an average increase of 3.21% in the root mean square prediction error relative to the naive algorithm in the simulation study. This is consistent with the differences reported in Table 3 for the data.

## 5. Simulation Study

### 5.1. Simulation Set-Up

The simulation study covers a wide range of scenarios, each evaluated using 30 replicates. Each replicate is constructed using the following process. Initially, the Spanish gross domestic product actual data to be predicted in our data analysis is used to obtain parameter estimates ($\hat{\mu }$, $\hat{\phi }$ and $\hat{\sigma }$), for a standard autoregressive process of order one, $y_{t}=\mu +\phi y_{t-1}+\sigma_{\epsilon }\epsilon_{t}$. These estimates are used in each replicate to generate a preliminary simulated target time series, $\left\{{\tilde{y}}_{t}^{*}\right\}$. This preliminary target is then used to generate simulated predictions for each institution, $\left\{{\tilde{y}}_{i,t}\right\}$, by adding noise (all noises considered, i.e., $\epsilon_{t}$, $\eta_{t}$ and $\varepsilon_{t}$), are independent standard Gaussian noises) with different intensities parameterized by its standard deviation, that is, ${\tilde{y}}_{i,t}={\tilde{y}}_{t}^{*}+\sigma_{\eta }\eta_{t}$. These simulated predictions are then aggregated using simulated weights, $\left\{{\tilde{\omega }}_{i}\right\}$. Simulated weights depend on the number of key agents (institutions) considered. For a 100% of key agents, simulated weights are set to equal weights. For 40% and 10% of key agents, that percentage of the total of institutions is randomly selected and randomly assigned uniform weights between 0.5 and 1. The other institutions are assigned a negligible weight and all weights are rescaled to add up to one. These simulated weights are used to produce the final simulated target time series ${\tilde{y}}_{t}=\sum_{i}{\tilde{\omega }}_{i}{\tilde{y}}_{i,t}+\sigma_{\varepsilon }\varepsilon_{t}$ (with $\sigma_{\varepsilon }$ fixed at 0.1 to introduce some but not much deviation from the direct aggregate). Algorithm performance for different such simulated target time series is analyzed by varying the following parameters: the number of institutions, the sample size, the percentage of key agents and the noise standard deviation.

The number of institutions or agents takes values 10, 20 and 40. The first two values are slightly under and slightly over the number of institutions in our data analysis (Section 4, Table 1). The third value corresponds to an ideal, large number of institutions. Sample size (T) takes values 6, 10 and 20. The first value matches the observations available in our data analysis and the other values consider reasonable and desirable horizons respectively. The percentages of key agents considered are 10%, 40% and 100%, with the latter corresponding to all institutions weighting equally in the generating the target time series. The noise standard deviation (SD), $\sigma_{\eta }$, takes values 0.1, 0.2 and 0.3. While the first two values are appropriate for near-future forecasts (e.g., forecasts for a given year made in December of that same year), the last value corresponds to forecasts further into the future (e.g., for a given year made in July of the preceding year).

### 5.2. Simulation Results

The results from the simulation study are as expected (Table 3 and Table 4). The proposed algorithm becomes preferable to the simpler, naive overall average as the length of the target time series increases and as the number of both institutions and key institutions decreases. The simulation study reveals that the root average square error can more than double when using the naive algorithm instead of the proposed one. Also, while the results show a good number of improvements of relative error over 20%, negative results seem to stop at around 12%.

The results in terms of weight recovery are shown in Table 4. We assess weight recovery via the Kullback–Leibler divergence between true and recovered weights. A small Kullback–Leibler divergence between these weights is linked to the improvements identified by the simulation study in forecast error resulting from applying the proposed algorithm. The results from Table 4 are in agreement with those from Table 3.

The so-called *forecast combination puzzle* consists in the realization that simple combinations of point forecasts have been found to outperform elaborated weighted combinations in repeated empirical applications [28]. Smith and Wallis [28] pointed out at finite-sample errors in weight estimation as a likely culprit. More recently, Genre et al. [13] establish that “we would not conclude that there exists a strong case for considering combinations other than equal weighting as a means of better summarizing the information collected as part of the regular quarterly rounds“ of the Survey of Professional Forecasters. Our findings are in agreement with this literature. The agreement is both from the empirical perspective and from that of the simulation study. This agreement complements the main contribution of this paper in connecting the information theory literature with the machine learning literature in the context of forecast combination. The success of equal weighting for forecast combination can also be linked to the fact that forecasting institutions tend to form a well-informed consensus, which benefits simultaneously from a herd effect [29] and a wisdom-of-the-crowds effect [30].

## 6. Concluding Remarks

According to prediction and sampling theories, forecasting errors and variances of single forecasts can be reduced by combining individual predictions. The traditional methods for combining forecasts are based on assessing the relative past performance of the forecasters to be combined. The problem, however, becomes indeterminate as soon as the number of forecasters is larger than the number of past results. To overcome this issue, an alternative is to assume some set of a priori weights and to apply the principle of maximum entropy to obtain a set of a posteriori weights, subject to the constraint that the combined predictions equal the realized values. Unfortunately, this is a complex problem that grows with the cardinality of the variables and the possibility of finding a solution is not guarantee.

In order to reach a solution within the information theory framework we propose a fresh approach to the problem and, inspired in the machine learning literature, we suggest a new specification based on regularization regression and an algorithm to solve it. The new approach always produces a solution, being moreover quite flexible. It permits the use of different norms to measure the discrepancies among the combined predictions and the realized values and to weight the relative importance of the discrepancies. Our regularization approach also has the advantage of producing, as a by-product, the weights assigned to the different forecasters. These weights could be understood as a measure of the forecasters’ ability and be used as a tool to decide the methodologies deserving more credit.

Further flexibility could be introduced in our model. For instance, by substituting in Equation (2) the single prediction values by prediction functions (for example, regression equations). In this case, the parameters of such prediction functions would be estimated simultaneously, during the cross-validation step. This will enable us to apply our proposal in one step when, for instance, we try to obtain, from a set of national forecasts, a prediction for a regional economy where single forecasts are not available. We could substitute the (unavailable) single regional forecasts for a parametrized function (e.g., a dynamic regression equation) of the national values.

In our algorithm, we have considered a quadratic norm (a ridge penalty) and a rolling-origin evaluation as cross-validation strategy. Obviously, other penalties (e.g., lasso or elastic net) are also possible and, likewise, there is also room for implementing other methods of cross-validation. For instance, we can explicitly omit the temporal order of the data in the training sets and carry out leave-one-out cross-validation. At the end, the relative importance of the most recent predictions can be implicitly included in our specification through the δ’s coefficients.

Regarding our application, as it is a common practice we have used the last reliable GDP available figures (all the countries elaborate several vintages of GDP. National accounts are regularly revised as statistical information is enlarged. For instance, in the case of Spain, the estimates from each year undergo three revisions until they are considered definite [31]) as realized values, $a_{t}$. In our opinion, this is not however the best strategy to be followed for a “combiner” of macroeconomic forecasts. Instead, flash estimates should be used. Flash estimates (the most provisional and least reliable figures, though) are the most appealing, getting a strong attention (on the one hand, they occupy the front pages of the media and are the ones more analysed, debated and commented on. Revised and definitive data, published three to four years later, attract little public opinion interest. On the other hand, and more importantly, the flash estimates serve as a framework for decision-making by economic stakeholders. Decisions which may give rise to rights and obligations: budgetary stability commitments in the EU, ceilings on general government expenditure, size of deficit or government debt allowed). This may entail marked consequences on the weights each forecaster receives.

The key contribution of this paper is to link the maximum-entropy inference methodology from the information theory literature with regularization from the machine learning literature with the ultimate goal of combining forecasts. Although one might envisage linking forecast combination algorithms other than regularization (e.g., boosting or bagging) with the information theory literature, it does not seem immediatly clear how this could be done. Such immediacy seems to be one of the advantages of regularization over alternative algorithms when it comes to connecting the machine learning and information theory literature.

## Figures and Tables

**Table 1:** Optimal weights $\omega_{i}^{*}$ output by the proposed algorithm.

Institucion	2002	2003	2004	2005	2006	2007	2008	2009	2010	2011	2012	2013	2014	2015	2016	2017	2018
Analistas Financieros	0.00	0.00	0.01	0.01	0.01	0.04	0.04	0.01	0.01	0.05	0.08	0.09	0.09	0.10	0.10	0.16	0.16
Bankia	0.00	0.00	0.01	0.01	0.01	0.04	0.04	0.01	0.01	0.05	0.08	0.08	0.08	0.07	0.08	0.11	0.11
BBVA	0.00	0.00	0.01	0.01	0.01	0.04	0.04	0.01	0.01	0.04	0.08	0.07	0.07	0.04	0.04	0.06	0.06
Caixabank	0.00	0.00	0.01	0.01	0.01	0.05	0.05	0.01	0.01	0.05	0.08	0.09	0.09	0.11	0.09	0.13	0.14
CatalunyaCaixa	1.00	1.00	0.03	0.04	0.06	0.17	0.17	0.36	0.37	0.33	0.08	0.10	0.10				
CEPREDE-UAM	0.00	0.00	0.01	0.01	0.01	0.05	0.05	0.01	0.01	0.04	0.08	0.07	0.07	0.04	0.04	0.05	0.05
Funcas	0.00	0.00	0.01	0.01	0.01	0.04	0.04	0.01	0.01	0.04	0.08	0.08	0.08	0.06	0.07	0.10	0.10
ICAE-UCM	0.00	0.00	0.01	0.01	0.01	0.03	0.03	0.01	0.01	0.04	0.08	0.08	0.08	0.06	0.05	0.07	0.07
ICO	0.00	0.00	0.13	0.64	0.74	0.26	0.26										
IEE	0.00	0.00	0.75	0.22	0.11	0.13	0.13	0.52	0.51	0.13	0.08	0.10	0.10	0.25	0.27		
Instituto Flores de Lemus	0.00	0.00	0.01	0.01	0.01	0.04	0.04	0.01	0.01	0.04	0.08	0.08	0.08	0.06	0.06		
Intermoney	0.00	0.00	0.01	0.01	0.01	0.04	0.04	0.01	0.01	0.08	0.08	0.08	0.08	0.08	0.09	0.12	0.12
Santander	0.00	0.00	0.02	0.02	0.02	0.07	0.07	0.03	0.03	0.12	0.08	0.09	0.09	0.12	0.12	0.19	0.19

**Table 2:** Gross Domestic Product (GDP), forecasts ${\hat{a}}_{T+1}$ and corresponding sample forecast root mean square errors (RMSE) for the time period 2000–2018 using different methods: the arithmetic average of predictions made by all institutions (naive); the proposed algorithm (machine); and the arithmetic average of the subset of predictions used to make the predictions with the proposed algorithm (naive2).

Year	GDP	Naive	Naive2	Machine
2000	5.20	4.01		
2001	4.00	3.00		
2002	2.90	2.09	2.01	2.30
2003	3.20	2.28	2.22	2.10
2004	3.20	2.81	2.77	2.74
2005	3.70	3.28	3.29	3.28
2006	4.20	3.37	3.39	3.30
2007	3.80	3.85	3.88	3.84
2008	1.10	1.74	1.69	1.76
2009	−3.60	−3.64	−3.57	−3.65
2010	0.00	−0.59	−0.52	−0.72
2011	−1.00	0.79	0.80	0.86
2012	−2.90	−1.69	−1.56	−1.60
2013	−1.70	−1.49	−1.48	−1.50
2014	1.40	1.19	1.20	1.18
2015	3.60	3.05	3.03	3.09
2016	3.20	2.85	2.83	2.88
2017	3.00	3.15	3.16	3.16
2018	2.60	2.79	2.82	2.84
Sample RMSE		0.76	0.73	0.74

**Table 3:** Relative changes (in %) of root average square error (averaging over years) of the arithmetic average of simulated institution predictions (“naive2” in Table 2) with respect to the the proposed algorithm. The parameters are the number of institutions or agents, sample size T (inner subtable dimensions), key agents and noise standard deviation (outer dimensions).

	Key			Noise SD=0.1					Noise SD=0.2					Noise SD=0.3		
	Agents			Sample Size (T)					Sample Size (T)					Sample Size (T)		
				
			Agents	T=6	T=10	T=20			T=6	T=10	T=20			T=6	T=10	T=20
			10	9.054	4.412	18.990			30.949	38.425	54.184			47.125	104.228	103.081
	10%		20	−10.597	−4.592	−1.951			0.189	6.892	9.293			11.791	21.052	34.796
			40	−2.586	−5.273	−3.923			−2.806	−1.578	−0.214			−1.774	0.725	2.796
				
			Agents	T=6	T=10	T=20			T=6	T=10	T=20			T=6	T=10	T=20
			10	−7.664	−7.808	−3.638			−1.210	−3.582	−3.214			2.117	0.619	4.131
	40%		20	−9.622	−0.997	−4.204			−10.501	−7.674	−6.759			−8.960	−7.862	−4.530
			40	−6.705	−3.98	−6.479			3.712	−1.271	−6.835			−4.716	−5.989	−3.499
				
			Agents	T=6	T=10	T=20			T=6	T=10	T=20			T=6	T=10	T=20
			10	−5.369	−5.737	−6.963			1.667	−10.27	−6.739			−9.857	−9.534	−8.856
	100%		20	−11.972	−8.850	−3.873			−9.304	−12.018	−7.438			−11.344	−8.470	−6.108
			40	−10.179	−9.636	−5.620			−11.407	−8.597	−3.475			−9.157	−7.703	−7.479

**Table 4:** Kullback–Leibler divergence between true and recovered weights.

	Key			Noise SD=0.1					Noise SD=0.2					Noise SD=0.3		
	Agents			Sample Size (T)					Sample Size (T)					Sample Size (T)		
																
			Agents	T=6	T=10	T=20			T=6	T=10	T=20			T=6	T=10	T=20
			10	2.653	1.197	0.362			0.815	0.376	0.154			0.686	0.166	0.116
	10%		20	2.964	2.378	1.438			1.958	1.316	0.574			1.663	0.930	0.286
			40	3.118	2.623	2.253			2.483	2.314	1.832			2.122	1.960	1.295
																
			Agents	T=6	T=10	T=20			T=6	T=10	T=20			T=6	T=10	T=20
			10	2.495	1.613	1.229			1.429	1.015	0.702			1.229	0.734	0.438
	40%		20	2.638	2.232	1.143			1.497	1.083	0.960			1.208	0.986	0.871
			40	2.053	1.462	1.178			1.281	1.220	1.022			1.039	1.163	0.960
																
			Agents	T=6	T=10	T=20			T=6	T=10	T=20			T=6	T=10	T=20
			10	1.647	1.346	0.357			0.639	0.356	0.068			0.371	0.253	0.035
	100%		20	1.173	0.860	0.613			0.637	0.259	0.098			0.222	0.135	0.092
			40	0.856	0.731	0.392			0.423	0.304	0.325			0.294	0.294	0.155
